# Genome-Wide Analysis of Known and Potential Tetraspanins in *Entamoeba histolytica*

**DOI:** 10.3390/genes10110885

**Published:** 2019-11-03

**Authors:** Kentaro Tomii, Herbert J. Santos, Tomoyoshi Nozaki

**Affiliations:** 1 Artificial Intelligence Research Center, National Institute of Advanced Industrial Science and Technology (AIST), 2-4-7 Aomi, Koto-ku, Tokyo 135-0064, Japan; 2 Department of Biomedical Chemistry, Graduate School of Medicine, The University of Tokyo, 7-3-1 Hongo, Bunkyo-ku, Tokyo 113-0033, Japan; hjsantos@m.u-tokyo.ac.jp

**Keywords:** tetraspanin, *Entamoeba*, membrane protein, subcellular localization, intron

## Abstract

Tetraspanins are membrane proteins involved in intra- and/or intercellular signaling, and membrane protein complex formation. In some organisms, their role is associated with virulence and pathogenesis. Here, we investigate known and potential tetraspanins in the human intestinal protozoan parasite *Entamoeba histolytica*. We conducted sequence similarity searches against the proteome data of *E. histolytica* and newly identified nine uncharacterized proteins as potential tetraspanins in *E. histolytica*. We found three subgroups within known and potential tetraspanins, as well as subgroup-associated features in both their amino acid and nucleotide sequences. We also examined the subcellular localization of a few representative tetraspanins that might be potentially related to pathogenicity. The results in this study could be useful resources for further understanding and downstream analyses of tetraspanins in *Entamoeba*.

## 1. Introduction

The human intestinal parasite, *Entamoeba histolytica*, is a pathogen that causes amoebiasis. This disease is characterized by colitis, often manifested by symptoms such as diarrhea and dysentery. It affects millions worldwide and causes up to 73,800 deaths annually [1]. Extraintestinal complications of amoebiasis arise when the parasite establishes colonization of other organs such as the liver, leading to amoebic liver abscess formation. The virulence of this parasite is due to its ability to inflict damage to host cells and tissues, which involve processes that are directly caused or regulated by membrane proteins. Among them are the galactose and N-acetyl-d-galactosamine specific lectin [2], adhesin [3], and the lysine and glutamic acid enriched protein (KERP1) [4], involved in parasite attachment to colonic epithelial cells, and rhomboid protease [5], which contribute to immune evasion.

Tetraspanins are membrane proteins with four transmembrane helices, two extracellular domains, and three short intracellular regions. They possess a ‘CCG’ motif, which is part of the well-conserved disulfide bonds in their extracellular domain 2 (EC2). Tetraspanins play important roles in organizing various cell-to-cell interactions and signal pathways [6]. Some tetraspanins are capable of forming stable interactions with other proteins such as receptor and signaling proteins, including other tetraspanins, leading to the establishment of functional complexes in the plasma membrane called tetraspanin microdomains [7]. Some of the known tetraspanins are integrated to the membranes of extracellular vesicles including CD9, CD63, CD81, CD82, and CD151 [8]. Mammalian tetraspanins have also been found to be involved in immune cell migration [9], in controlling membrane architecture [10], in cancer cell invasion and metastasis [11], as well as in mediating viral and non-viral membrane fusions [12].

Among parasites, tetraspanins have been reported to be involved in tegument formation of some helminths such as *Opisthorchis viverrini* [13] and *Schistosoma mansoni* [14] and are promising candidates for vaccine development [15]. In the unicellular parasite *Trichomonas vaginalis*, the tetraspanin TvTsp1 was found to be associated to exosomes [16] while TvTsp6, localized in the flagella, is involved in sensory reception and migration during infection [17]. In addition to the reported pathogenesis-associated membrane proteins in *Entamoeba*, it is likely that tetraspanins may also play a role in infection and virulence, however they have not been well characterized so far. Thus, we performed a genome-wide analysis to elucidate and scrutinize potential tetraspanins in *E. histolytica* and in other species of *Entamoeba*. Here, we provide a refined list of tetraspanins including both known and potential ones revealed by our bioinformatic analysis of the proteome in *E. histolytica* and in four other species of genus *Entamoeba*. We analyzed the evolutionary relationships of the candidate tetraspanins, as well as examined the subcellular localization of a few representative candidates. The list and results in this study could be useful resources for further understanding and analyses of tetraspanins in *Entamoeba*.

## 2. Materials and Methods

### 2.1. Sequence Data of Entamoeba histolytica and Other Entamoeba

As a reference of amino acid sequences, we used the proteome of *Entamoeba histolytica* (Strain: ATCC 30459/HM-1: IMSS; Proteome ID: UP000001926), consisting of 7959 proteins, provided by UniProt [18]. In this study, we regarded eight proteins (see Results and Table 1) reported in previous studies [19,20] as “known” *E. histolytica* tetraspanins. Nucleotide sequences, including introns, of tetraspanin genes in *E. histolytica* and in four other *Entamoeba* species (i.e., *E. dispar*, *E. invadens*, *E. moshkovskii* and *E. nuttalli*) and amino acid sequences of tetraspanins in other *Entamoeba* were retrieved from AmoebaDB (41 Released) [21].

For the four tetraspanin sequences in *E. histolytica*, we used the alternative sequences (UniProtKB accession numbers (ACs): A0A175JUA3 (for TSPAN3), A0A175JDI1 (for TSPAN11), A0A175JRI8 (for TSPAN6) and A0A175JZG7 (for TSPAN17)), instead of the original ones as they likely lack their N-terminal portions. We used C4LYT6 (for TSPAN10) whose length is 197 amino acids, instead of A0A175JJX9 (191 amino acids), and used A0A175JXX9 (for TSPAN13) whose second residue is Ala, instead of C4M9J5 (for EHI_107790) whose second residue is Pro. The information on these entries can be found in Table 1.

### 2.2. Computational Analysis of Sequence Data

To detect uncharacterized but potential tetraspanins in *E. histolytica*, we performed similarity searches using the FASTA36 package [22] with an efficient substitution matrix, MIQS [23,24], which outperforms conventional matrices in terms of both detection sensitivity and alignment quality. For amino acid sequence comparisons, we used *ssearch36* with an expectation threshold of 0.001. Filtering low-complexity regions of amino acid sequences by the PSEG program [25] was done using the University of Virginia (UVA) FASTA server [26]. To compare amino acid sequences to the nucleotide sequences, we used tfastx36/tfasty36 in the package.

We used FAMSA (ver. 1.2.5) [27], which also used MIQS as a default matrix, to construct multiple sequence alignments of tetraspanins. We performed the neighbor-joining method using the JTT substitution model with the “estimate” option for heterogeneity among sites, at the MAFFT server [28], based on a multiple sequence alignment to estimate a phylogenetic tree. We used, as an outgroup protein, a putative trans 2,3-enoyl-coa reductase, C4LTB8 (CL6EHI_045030) with four transmembrane helices. For phylogenetic tree calculations, we used only the gap-free regions (151 residue positions in total) found in the multiple sequence alignment. We resampled 1000 times for calculating the neighbor-joining bootstrap support values. We used RNAfold (Version 2.4.8) [29] with a default setting to predict the secondary structure of intronic RNAs.

### 2.3. Amoeba Culture and Plasmid Construction

Trophozoites of *Entamoeba histolytica* HM-1: IMSS clone 6 [30] were axenically cultured using Diamond BI-S-33 medium [30]. Genomic DNA was isolated by first washing the cells with 1 x phosphate-buffered saline (PBS), followed by lysis using cell lysis buffer C1 (Qiagen, Hilden, Germany). The resulting lysate was centrifuged at 1300× *g* for 10 min at 4 °C. The pellet containing nuclei was washed and reacted in ice cold buffer C1 before centrifugation at 1300× *g* for 10 min at 4 °C. Buffer G2 (Qiagen, Hilden, Germany) was added to the washed pellet and thoroughly mixed by inversion, followed by treatment with Protease K (Qiagen, Hilden, Germany) and RNase (Qiagen, Hilden, Germany). The mixture was incubated for 1 h at 50 °C. Then, 1:1 TE-saturated phenol–chloroform was added followed by gentle mixing by inversion. The resulting mixture was incubated for 5 min on ice. Afterwards, the mixture was centrifuged at 8600× *g* for 10 min at 4 °C. The upper phase was transferred to a new tube and reacted with chloroform. The mixture was incubated for 2 min on ice, then centrifuged at 8600× *g* for 10 min at 4 °C. The upper phase was collected and treated with Pellet Paint (Novagen, Madison, WI, USA), 3 M sodium acetate (pH 5.2) and ethanol to precipitate DNA. The genomic DNA pellet was washed with 70% ethanol. After removal of residual ethanol, the pellet was dissolved in TE buffer before storage at −80 °C.

All polymerase chain reaction amplifications were performed using Tks Gflex DNA polymerase (Takara, Shiba, Japan). Primer sets used are summarized in Table S1. Amplified DNA fragments were digested by *Bgl*II (New England Biolabs, Beverly, MA, USA), and ligated into pEhEx-HA [31] using Ligation-Convenience Kit (Nippongene, Tokyo, Japan). Transfection by lipofection, selection, and maintenance of amoebic transformants were performed as previously described [32].

### 2.4. Immunoblot Analysis and Immunofluorescence Assay (IFA)

Trophozoites of representative hemagluttinin (HA)-tagged tetraspanin-expressing strains were lysed, and approximately 20 μg proteins were separated by denaturing sodium dodecyl sulfate-polyacrylamide gel electrophoresis (SDS-PAGE) [33]. A strain transfected with the pEhEx-HA empty vector was used as control. Proteins were transferred to polyvinylidene difluoride (PVDF) membranes and were stained with anti-HA monoclonal antibody (16B12, Biolegend, San Diego, CA, USA and anti-cysteine synthase 1 (CS1; an enzyme involved in sulfur-containing amino acid metabolism) antiserum (loading control) as described previously [34]. IFA was performed as previously described [32], with some modifications. Cells were double-stained with mouse anti-HA antibody and one of the following rabbit antisera; anti-adenosine-5’-phosphosulfate kinase (APSK) [35] as mitosome marker, anti-binding immunoglobin protein (BiP) [36] as ER marker, and anti-vacuolar protein sorting-associated protein 26 (Vps26) [37] as endosome marker, diluted 1:500 (except for anti-APSK antiserum, which was diluted 1:300), respectively in 0.2% saponin and 1% bovine serum albumin in PBS. Samples were visualized using a confocal laser scanning microscope, LSM780 (Carl Zeiss Microscopy, Oberkochen, Germany).

## 3. Results

### 3.1. Identification of Potential Tetraspanins in Entamoeba histolytica

Two articles which analyzed evolutionary relationships of tetraspanins including ones in *E. histolytica*, have been published so far [19,20], both of which reported six candidate *E. histolytica* proteins as tetraspanins. Among those proteins, two [C4LUK2 (TSPAN1 in this study) and C4M992 (TSPAN4)] were reported in both studies. One of the proteins used in the former study [19], EAL45935 (EHI_139370; UniProtKB AC: C4M6W3) is a high mobility group (HMG) box domain containing protein, according to the annotation provided by UniProt, and contains only a single cysteine residue. In addition, we could not find any statistically significant similarity between the protein and a plausible tetraspanin in *E. histolytica*. Thus, we did not regard C4M6W3 as a candidate tetraspanin in this study. One of the proteins reported in the later study [20], EHI5A_058850 (EHI_127260; UniProtKB AC: C4LWZ8) is a Plecksin homology domain protein, according to the annotation by UniProt, and lacks the CCG motif. Thus, we also excluded this protein as a candidate tetraspanin in *E. histolytica*, though we found weak but significant similarity between this protein and C4M9D7 (TSPAN12).

Using *ssearch36* with the MIQS matrix and the latest proteome of *E. histolytica*, we could identify nine additional uncharacterized proteins as potential tetraspanins in *E. histolytica* in addition to the eight remaining proteins derived from the previous studies. Table 1 lists the refined set of 17 proteins including both known and potential tetraspanins in *E. histolytica*. The lengths of the proteins are around 200 (or more) amino acids and are similar to each other (191 to 225 amino acids), except for two, TSPAN5 (259 aa) and TSPAN17 (254 aa). The proteins listed in Table 1 have been annotated to possess four transmembrane helices by Phobius [38] in Pfam [39], except for five proteins, TSPAN3, 6, 11, 13, and 17. However, they are not registered as a member of the tetraspanin family (PF00335) in Pfam, except for one, TSPAN4 (/C4M992).

### 3.2. Possible Subgroups of Entamoeba Tetraspanins

**Figure 1:**
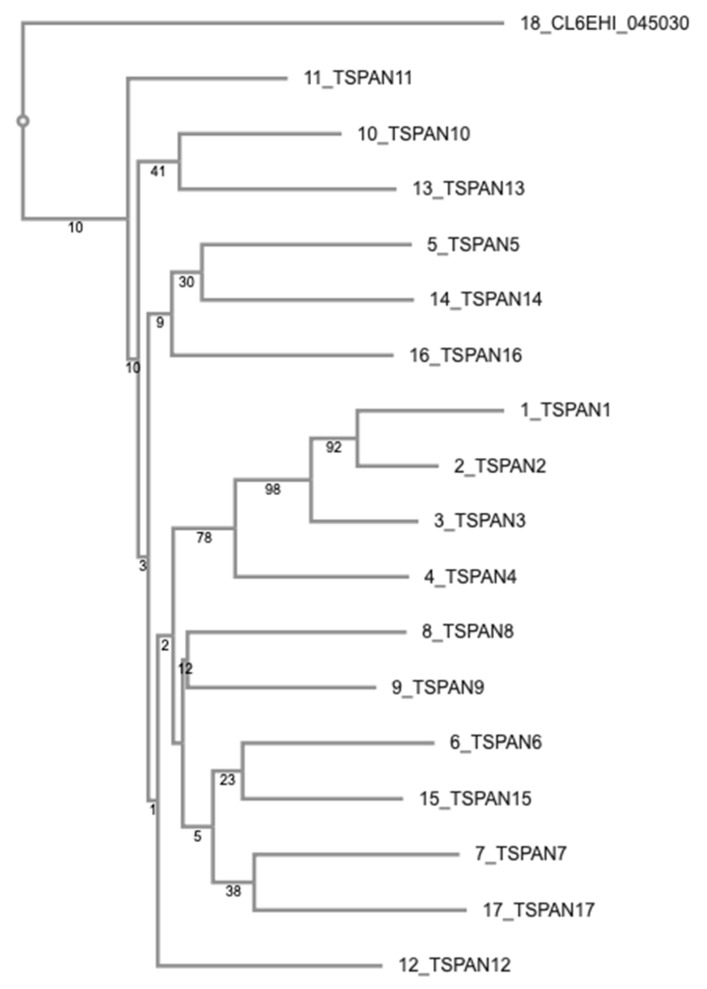
Phylogenetic tree of 17 tetraspanins in *E. histolytica*. Bootstrap support values are placed at each branch point.

**Figure 2:**
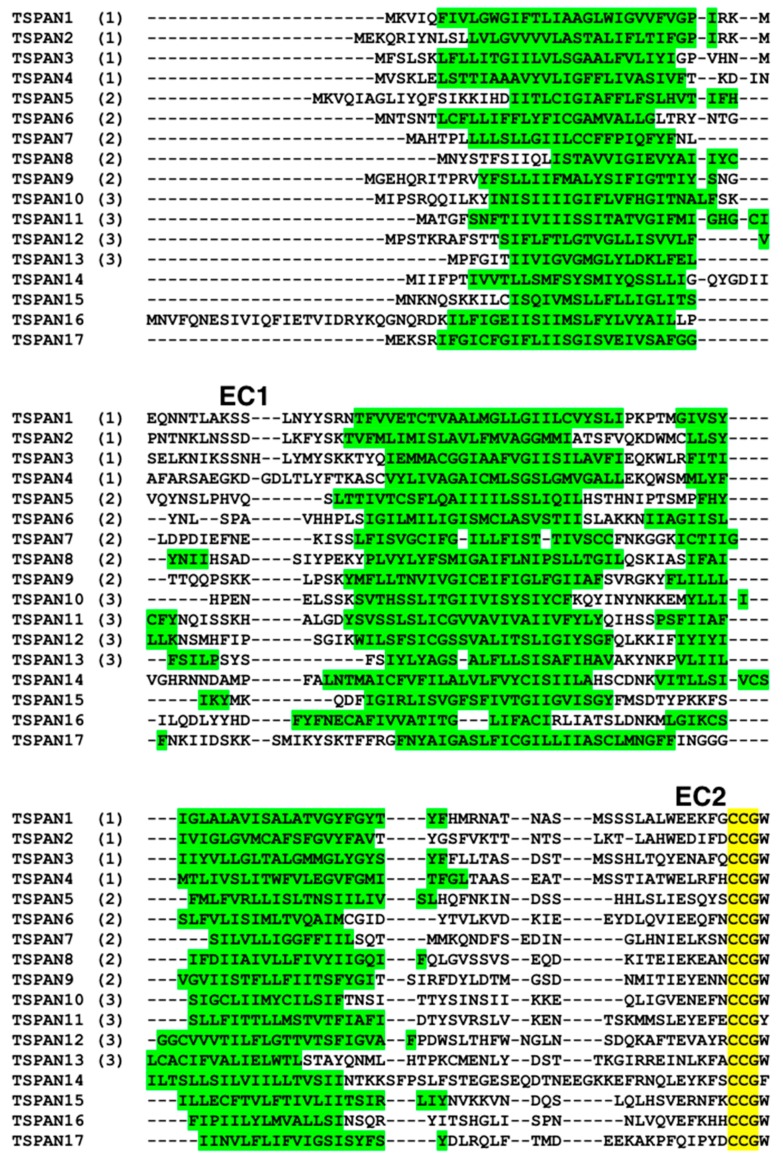

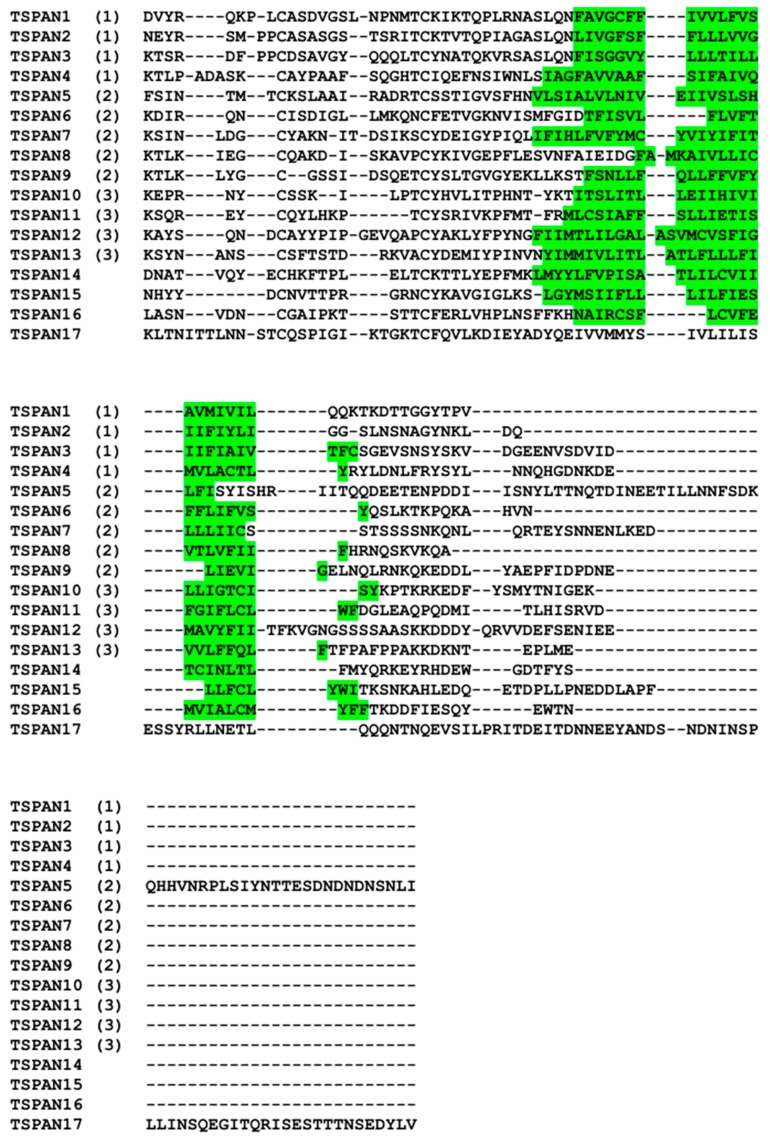
Multiple sequence alignment of 17 tetraspanins in *E. histolytica*. Putative membrane-spanning regions provided by UniProt are in green. The CCG motifs are highlighted in yellow. Locations of EC1 and EC2 are displayed respectively. Numbers in parentheses indicate subgroup numbers.

We tried to analyze evolutionary relationships of the 17 proteins in Table 1. Most of them are highly diverged so that evolutionary relationships among them are obfuscated. Figure 1 shows a neighbor-joining tree based on a multiple sequence alignment of the 17 proteins and C4LTB8 (CL6EHI_045030), as an outgroup, calculated by FAMSA (Figure 2). Although the neighbor-joining bootstrap support values are not so high in most cases, we were able to recognize, to some extent, characteristic residues that appear to be associated within certain subgroups (Table 2).

**Figure 3:**
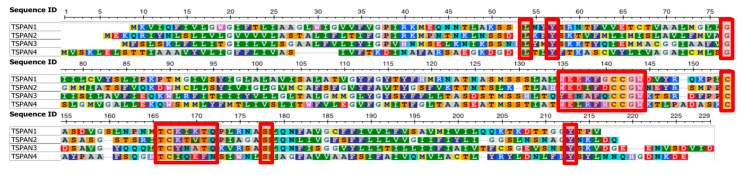
Multiple sequence alignment of subgroup 1 tetraspanins in *E. histolytica*. Conserved residues and regions mentioned in the main text are indicated with red boxes. The figure was generated using the NCBI MSA Viewer [40].

The result of our phylogenetic analysis suggests that there are several clades of tetraspanins in *E. histolytica*, one of which appears to have diverged relatively recently from others. This subgroup (subgroup 1) consists of TSPAN1, TSPAN2, TSPAN3, and TSPAN4. These four proteins are relatively conserved within this subgroup, compared with the other tetraspanins in *E. histolytica*. We found conserved residues associated with this subgroup (Figure 3). Among the four proteins, “ΩExxFxCCGW–C–TCxxxx [Q/N]”, where Ω and x represent aromatic and any types of amino acids respectively, around the CCG motif in EC2 is completely conserved. In addition, Leu and Tyr in extracellular domain 1 (EC1), Gly in the second transmembrane helix, Ser at the end of EC2, and Tyr in the C-terminal intracellular regions, are uniquely conserved among the four proteins. These characteristics of having shared residues in proteins belonging to subgroup 1 are almost conserved among orthologous proteins in four other *Entamoeba* species (i.e., *E. dispar*, *E. invadens*, *E. moshkovskii* and *E. nuttalli*) (Figure S1), thus, they might have implications to their function.

Characteristic residues are also found in the other subgroups of *Entamoeba* candidate tetraspanins. We defined “Subgroup 2”, comprising TSPAN5, TSPAN6, TSPAN7, TSPAN8, and TSPAN9 (Table 2), based on the uniquely conserved sequence in their EC2. The proteins in subgroup 2, as well as their orthologous proteins in *E. dispar*, *E. invadens,* and *E. nuttalli*, possess an almost conserved sequence of “IExxx [N/S]CCGW–C–CxxxxG” in their EC2, though some proteins in *E. nuttalli* have slightly different sequences from this pattern (Figure S2). Although TSPAN15 and TSPAN17 are in the same clade with the proteins in subgroup 2 based on the phylogenetic tree (Figure 1), they lack this sequence pattern. Moreover, TSPAN15 might be related to subgroup 3, as discussed in Section 3.3 below. TSPAN5 is not in the same clade with proteins of this subgroup, however, it possesses the same sequence pattern and its gene contains no introns (see Section 3.3). Thus, we tentatively regarded TSPAN5 as a member of subgroup 2.

A third subgroup named “Subgroup 3” consisting of four proteins, TSPAN10, TSPAN11, TSPAN12, and TSPAN13, was formed because these proteins do not possess “IE” but instead share “CY” in EC2. The four proteins in subgroup 3, and their orthologs in the other four species of *Entamoeba* share a completely conserved segment in their EC2, with the following sequence “[E/N]xx[F/Y]xCCGΩK–C–CYxx[I/L/M]xxP”. Also charged residues tend to be clustered in the C-terminal intracellular regions among the subgroup 3 proteins (Figure S3).

### 3.3. Gene Structure of Tetraspanins in Entamoeba histolytica

Out of the 17 genes which potentially encode tetraspanins in *E. histolytica*, 10 were identified to contain intron(s). Among them, eight genes consist of one intron and two exons, while two genes have two introns and three exons. All 12 introns in the 10 genes have 5′GT/AG3′ splice sites, as well as the strongly conserved 5′ sequence, 5′GTTTG [41]. These gene structures are almost conserved within *Entamoeba*, except for *E. invadens* (Table 3 and Discussion Section).

The genes encoding proteins of subgroup 3 tend to possess more introns than others. In fact, EHI_091490 (TSPAN12) has two introns and three exons. Some orthologous genes of EHI_152810 (TSPAN11) and EHI_107790 (TSPAN13) in other *Entamoeba* also have two introns and three exons, though EHI_152810 and EHI_107790 only have a single intron. Note that alternative sequences of these genes are not registered in AmoebaDB, (CL6EHI_152810/TSPAN11 and CL6EHI_107790/TSPAN13) but are deposited in UniProtKB are longer. Hence, it is likely that these two genes also have at least one more intron in their longer forms. The rest of subgroup 3, EHI_010390 (TSPAN10), and its orthologous genes in *Entamoeba* have one intron and two exons.

**Figure 4:**
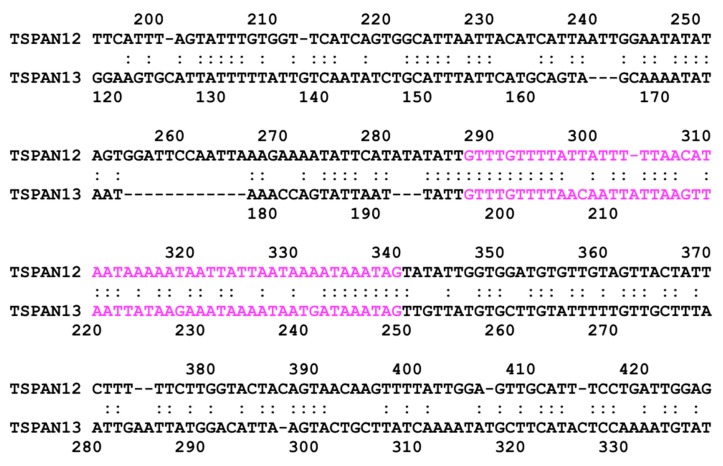
Local alignment of nucleotide sequences between TSPAN12 and TSPAN13 in *E. histolytica*. Letters in magenta represent intron sequences.

**Figure 5:**
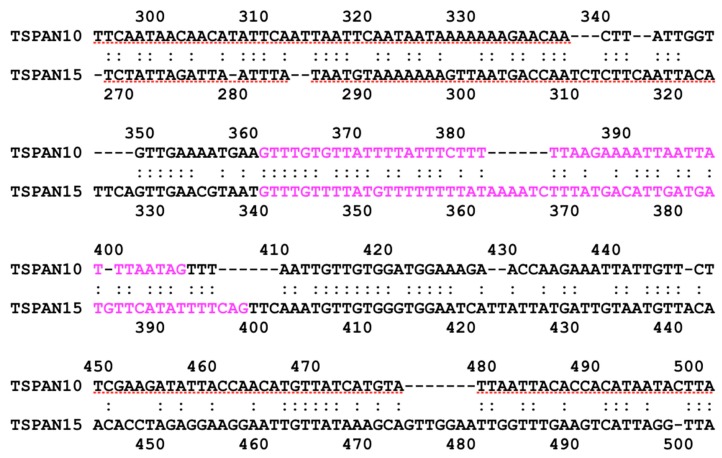
Local alignment of nucleotide sequences between TSPAN10 and TSPAN15 in *E. histolytica*. Letters in magenta represent intron sequences.

We also found sequence similarity between introns of subgroup 3 genes in *E. histolytica*. The second intron of EHI_091490 (TSPAN12) and the intron of EHI_107790 (TSPAN13) show over 70% identity at the corresponding position, i.e., at N-terminal regions of the third transmembrane helix, of both TSPAN12 and TSPAN13 (Figure 4). In addition, we can see similar relationships between EHI_010390 (TSPAN10) and EHI_016390 (TSPAN15). Their introns, which show sequence similarity, are located at the same position encoding three/two amino acids before the CCG motif in both genes (Figure 5), though TSPAN15 is in the same clade with proteins in subgroup 2 in the phylogenetic tree. These results suggest relatively close relationships between proteins in subgroup 3 (and also TSPAN15), though their amino acid sequences are highly diverged.

### 3.4. Subcellular Localization Analysis of Representative Tetraspanin Candidates

**Figure 6:**
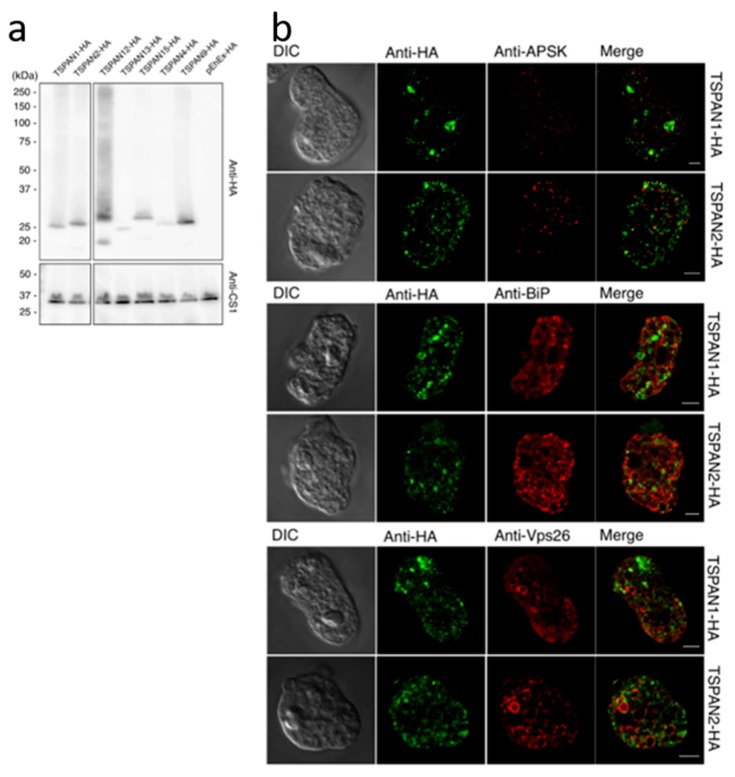
Expression of TSPAN1-HA and TSPAN2-HA in *E. histolytica.* (**a**) Immunoblot analysis of total cell lysate from representative tetraspanin-expressing and pEhEx-HA control strains respectively. Approximately 20 μg of protein was separated by SDS-PAGE and transferred to PVDF membranes. The membranes were reacted with anti-HA antibody (top panel) and anti-CS1 antiserum (bottom panel, loading control) respectively. (**b**) Immunofluorescence assay micrographs of TSPAN1-HA and TSPAN2-HA double-stained with mouse anti-HA antibody (green) and rabbit anti-APSK (red, top panel), anti-BiP (red, middle panel), and anti-Vps26 (red, bottom panel) antisera respectively. Scale bar, 5 µm.

We expressed a few representative tetraspanin candidates in *E. histolytica* trophozoites, with hemaglutinnin (HA) tag at the carboxyl-terminus (Figure 6a), including two proteins belonging to subgroup 1, TSPAN1 and TSPAN2, which are similar to each other (~35% identity in 200 aa). Both were originally found in the proteome of the mitosome, a class of mitochondrion-related organelle present in *Entamoeba* [32]. To verify the localization of these proteins, we performed immunofluorescence assay. Micrographs of cells stained with anti-HA antibody showed mostly vesicular signals of TSPAN1 and TSPAN2 (Figure 6b), rather than the punctate signal pattern associated with mitosome localization [32,42,43,44,45,46,47]. The anti-HA signals did not colocalize with the mitosome marker (anti-APSK: mitosomal matrix enzyme, [35]) in double-stained cells (Figure 6b, top panel), suggesting TSPAN1 and TSPAN2 are not localized to mitosomes. We then assessed whether the anti-HA signals colocalize with either ER (anti-BiP; ER lumen chaperone, [36]) or endosome (anti-Vps26: retromer-like complex component, [37]) markers (Figure 6b, middle and bottom panels respectively), however the immunofluorescence micrographs likewise suggest that TSPAN1 and TSPAN2 are not localized to either the ER or endosomes. Although the signals of TSPAN1 and TSPAN2 did not show colocalization with the organellar markers used, both proteins similarly showed mostly vesicular patterns suggesting they are localized to certain vesicular compartments in the cytosol. We also performed IFA using trophozoites that were expressing other representative tetraspanins (Figure S5). The anti-HA micrographs show a variety of anti-HA signal patterns, suggesting these candidate amoebic tetraspanins localize to different compartments in the cell. 

Another previously reported amoebic tetraspanin is TSPAN4 (C4M992), which was identified as differentially more highly expressed in the non-pathogenic A1^np^ strain than the pathogenic B2^p^ cell line. In addition, overexpression of TSPAN4 in B2^p^ clones caused a loss of the pathogenic phenotype, based on the significantly reduced amoebic liver abscesses formed when injected to gerbils, compared with the respective controls [48]. Association of TSPAN4 to subgroup 1, indicates that the other proteins in this cluster namely TSPAN1, 2, and 3, may also be potentially related with pathogenicity.

Meanwhile, the expression level of almost all candidate tetraspanins did not significantly change (≤2.0-fold change) when trophozoites were subjected to different stress conditions such as glucose starvation [49], cysteine deprivation [50], and paraquat treatment [51] as previously reported in three independent transcriptomic studies. However, we found that TSPAN17 was marginally upregulated (2.05-fold and 2.14-fold) after 24 h and 48 h of cysteine deprivation [50], respectively, and TSPAN7, was slightly upregulated (2.42-fold) after 12 h of paraquat treatment [51]. These data may suggest possible involvement of TSPAN17 and TSPAN7 in response to cysteine-depletion and oxidative stress, respectively. Interestingly, TSPAN7 and TSPAN17 are neighbors in the phylogenetic tree (Figure 1).

## 4. Discussion

Based on our computational analysis of proteomic and genomic data, we showed that *E. histolytica* possesses at least 17 putative tetraspanins, though the similarity search method used in this study is classical (but effective) [23,24]. We suppose that examining whether those proteins are tetraspanins or not is an important issue to be addressed. Although we have not experimentally verified functions of the potential tetraspanins, one (TSPAN16/C4M5N8) of them shows weak similarity (~18% identity in 185 aa) to TvTSP8, a tetraspanin that mediates parasite–parasite interactions in *Trichomonas vaginalis* [20].

According to the classification results of extracellular domain 2 (EC2) in the previous study [19], all 17 proteins in *E. histolytica* are the ancient 4c type. We found that there are three possible subgroups of tetraspanins in *E. histolytica*, and that uniquely conserved residues are present within each subgroup. Such residues are mainly clustered around the CCG motif in EC2. These may reflect the involvement of different interacting partners of each subgroup. Even so, the result of our phylogenetic analysis is not so robust, as tetraspanins in *E. histolytica* are highly diverged. In fact, phylogenetic trees can vary, except for subgroup 1, depending on MSAs, parameters, and methods used in calculations. Therefore, classification of tetraspanins in *E. histolytica* may be updated or improved with further analysis.

It is also plausible that the C-terminal intracellular regions of potential tetraspanins in *E. histolytica* may have crucial roles related to their subcellular localization. In this study, we found three distinctive patterns in the C-terminal regions of potential tetraspanins. In subgroup 1, Tyr in the C-terminal regions is uniquely conserved. We recognize similarity between a sequence around this tyrosine and a tyrosine-based sorting signal, YxxΦ, where Φ represents a bulky hydrophobic amino acid [52]. Leu/Val occurred at the corresponding position to Φ in tetraspanins of subgroup 1. This tyrosine-based signal is involved in targeting to proteins of various intracellular compartments. The position, i.e. location at the C-terminal region of a protein, and distance from the membrane of YxxL/V are likely to be suitable for such a signal.

In subgroup 3, we found a cluster of charged residues, consisting of two or three positively charged residues and negatively charged residue(s), in their C-terminal regions. However, the functional implications of these conserved residues are not known. In addition, recently, it was reported that the C-terminal regions of tetraspanins in *Trichomonas vaginalis* regulate their subcellular localization [20]. As compared to the wild type, three *T. vaginalis* tetraspanins (TvTSP2, TvTSP3 and TvTSP5) show different localization upon truncation of the C-terminal region that includes a stretch of Ser residues. Two tetraspanins in *E. histolytica* also possess a similar stretch: 200SSSSAAS206 in TSPAN12 (C4M9D7) and 175SSTSSSS181 in TSPAN7 (C4M1M8), though this is a protein of subgroup 2. These portions in the C-terminal regions of potential tetraspanins in *E. histolytica* may likewise contain information related to their subcellular localization.

Our analysis of introns in *Entamoeba* tetraspanins showed that the members of subgroup 1, especially TSPAN1 and TSPAN2 are similar to each other, suggesting that these genes were relatively recently diverged from a common ancestor. However, only the TSPAN2 gene has an intron. Secondly, according to the previous study of 14 introns in *E. histolytica*, the average length was determined to be 65 nucleotides [53]. In this study we found that the average length among a total of 12 introns in the 10 candidate tetraspanin genes in *E. histolytica*, is about 53 nucleotides. Specifically, the average intron lengths among different *Entamoeba* species are 49 (from 11 introns in *E. dispar*), 53 (fromr 13 introns in *E. moshkovskii*), and 54 (from 11 introns in *E. nuttalli*) nucleotides, respectively, which is consistent with the previous observation. However, the number and length of introns of potential tetraspanin genes in *E. invadens* appear to be higher (18 introns) and longer (89 nucleotides on average) than in other species. These observations suggest that frequencies of intron generation (or elimination) in *Entamoeba* may be higher than previously considered [53] and may indicate the most recent divergence of *E. invadens* in *Entamoeba*. As shown above, there is a striking similarity between the introns of EHI_091490 (TSPAN12) and EHI_107790 (TSPAN13). We also found that these two similar introns can possess secondary structures (Figure S4), suggesting that there may be a relationship between the pre-mRNA secondary structure and splicing, as previously reported [54].

## 5. Conclusions

Based on sequence similarities to ‘known’ tetraspanins, we newly identified nine uncharacterized proteins as potential tetraspanins and provided a refined list of 17 tetraspanins in *E. histolytica*. Computational analysis of those sequences revealed existence of possible subgroups, and characteristic features associated with each subgroup contained in both amino acid and nucleotide sequences. Such features may have implications on the function and evolution of tetraspanins in *E. histolytica*.

## Figures and Tables

**Table 1:** A list of known and potential tetraspanins in *E. histolytica*.

Gene Name(s)	Entry Name (UniProtKB)	Length (aa)	in Previous Studies ^1^	Names in This Study
EHI_010390	C4LYT6_ENTHI	197	–	TSPAN10
EHI_016390 (CL6EHI_016390)	C4M3W1_ENTHI	196	EHI5A_042920	TSPAN15
EHI_022890 (CL6EHI_022890)	C4LUK2_ENTHI	206	EAL48097 EHI5A_127460	TSPAN1
EHI_040610 (CL6EHI_040610)	C4M5N8_ENTHI	213	–	TSPAN16
EHI_055360 (CL6EHI_055360)	C4M9Q1_ENTHI	222	EHI5A_158290	TSPAN14
EHI_075690 (CL6EHI_075690)	C4M992_ENTHI	218	EAL43389 EHI5A_106540	TSPAN4
EHI_091490 (CL6EHI_091490)	C4M9D7_ENTHI	225	–	TSPAN12
EHI_097600 (CL6EHI_097600)	C4M931_ENTHI	209	–	TSPAN9
CL6EHI_107790	A0A175JXX9_ENTHI	195	EHI5A_153470	TSPAN13
EHI_133990 (CL6EHI_133990)	C4M1M8_ENTHI	200	–	TSPAN7
CL6EHI_139380	A0A175JUA3_ENTHI	218	–	TSPAN3
EHI_147520 (CL6EHI_147520)	C4M6M8_ENTHI	259	EAL49140	TSPAN5
CL6EHI_152810	A0A175JDI1_ENTHI	201	–	TSPAN11
EHI_164840 (CL6EHI_164840)	C4M1X7_ENTHI	191	–	TSPAN8
CL6EHI_168280	A0A175JRI8_ENTHI	193	–	TSPAN6
EHI_174220 (CL6EHI_174220)	C4M269_ENTHI	208	EAL47116	TSPAN2
CL6EHI_198000	A0A175JZG7_ENTHI	254	(EAL42794)	TSPAN17

^1^ IDs used in the two previous studies [19,20].

**Table 2:** Subgroups and motifs of 17 tetraspanins in *E. histolytica*.

TSPAN Name	Entry Name (UniProtKB)	Sequence Motifs					Subgroup
		IE	FxCCG	CCGW	TC	CY	
TSPAN1	C4LUK2_ENTHI	×	○	○	○	×	1
TSPAN2	C4M269_ENTHI	×	○	○	○	×	1
TSPAN3	A0A175JUA3_ENTHI	×	○	○	○	○	1
TSPAN4	C4M992_ENTHI	×	○	○	○	×	1
TSPAN5	C4M6M8_ENTHI	○	YSCCG	○	○	×	2
TSPAN6	A0A175JRI8_ENTHI	○	○	○	NC	×	2
TSPAN7	C4M1M8_ENTHI	○	SNCCG	○	SC	○	2
TSPAN8	C4M1X7_ENTHI	○	ANCCG	○	PC	○	2
TSPAN9	C4M931_ENTHI	○	NNCCG	○	○	○	2
TSPAN10	C4LYT6_ENTHI	×	○	○	○	○	3
TSPAN11	A0A175JDI1_ENTHI	×	○	CCGY	○	○	3
TSPAN12	C4M9D7_ENTHI	×	YRCCG	○	PC	○	3
TSPAN13	A0A175JXX9_ENTHI	×	○	○	AC	○	3
TSPAN14	C4M9Q1_ENTHI	×	○	CCGF	○	×	–
TSPAN15	C4M3W1_ENTHI	×	○	○	NC	○	(3?)
TSPAN16	C4M5N8_ENTHI	×	HHCCG	○	○	×	–
TSPAN17	A0A175JZG7_ENTHI	×	YDCCG	○	○	×	(2?)

×: absent, ○: present.

**Table 3:** Orthologous relationships and gene organizations of tetraspanins in *Entamoeba*.

Gene Name(s)/TSPAN Name	#Intron/Length (bp)	*E. dispar*	*E. invadens*	*E. moshkovskii*	*E. nuttalli*	Subgroup
		#Intron/Length (bp), Gene Name, Length (aa)				
EHI_022890 (CL6EHI_022890) TSPAN1	0/–	0/– EDI_248410 (206)	1/43 EIN_226110 (212)	0/– EMO_114210 (206)	0/– ENU1_002880 (206)	1
EHI_174220 (CL6EHI_174220) TSPAN2	1/53	1/51 EDI_018160 (208)	1/141 EIN_092430 (154)	1/56 EMO_051310 (208)	1/51 ENU1_208670 (208)	
CL6EHI_139380 TSPAN3	0/– ^1^	1/55 EDI_284040 (219)	1/42 EIN_227240 (189)	1/67 EMO_007720 (222)	0/– ENU1_174960 (184)	
EHI_075690 (CL6EHI_075690) TSPAN4	0/–	0/– EDI_076910 (218)	1/38 EIN_371310 (217)	0/– EMO_068760 (218)	0/– ENU1_204710 (218)	
EHI_147520 (CL6EHI_147520) TSPAN5	0/–	0/– EDI_063570 (259)	0/– EIN_164970 (287)	0/– EMO_039780 (269)	0/– ENU1_095870 (259)	2
CL6EHI_168280 TSPAN6	1/45 ^2^	0/– EDI_252640 (172)	0/– EIN_169070 (199)	1/42 EMO_099170 (193)	0/– ENU1_172260 (172)	
EHI_133990 (CL6EHI_133990) TSPAN7	0/–	–	0/– EIN_052950 (188)	0/– EMO_052920 (206)	0/– ENU1_202640 (201)	
EHI_164840 (CL6EHI_164840) TSPAN8	0/–	0/– EDI_282680 (191)	3/21, 77, 57 EIN_469620 (202)	0/– EMO_010630 (193)	0/– ENU1_142790 (191)	
EHI_097600 (CL6EHI_097600) TSPAN9	1/64	1/64 EDI_032730 (209)	1/171 EIN_228410 (179)	1/46 EMO_092880 (209)	1/64 ENU1_056490 (209)	
EHI_010390 TSPAN10	1/46	1/47 EDI_255180 (197)	–	1/44 EMO_033170 (199)	1/47 ENU1_058930 (197)	3
CL6EHI_152810 TSPAN11	1/55 ^3^	1/48 EDI_093040 (176)	2/83, 80 EIN_153280 (201)	2/52, 44 EMO_021620 (201)	1/54 ENU1_015090 (176)	
EHI_091490 (CL6EHI_091490) TSPAN12	2/50, 53	2/50, 54 EDI_217940 (226)	3/70, 148, 75 EIN_153850 (240)	1/44 EMO_103930 (192)	2/49, 53 ENU1_017640 (225)	
CL6EHI_107790 TSPAN13	1/54 ^4^	1/54 EDI_235170 (215)	2/261, 59 EIN_085580 (215)	1/48 EMO_090110 (215)	1/54 ENU1_021210 (215)	
EHI_055360 (CL6EHI_055360) TSPAN14	2/48, 55	1/55 EDI_044480 (209)	0/– EIN_224490 (222)	2/61, 40 EMO_001580 (222)	2/48, 58 ENU1_087800 (222)	–
EHI_016390 (CL6EHI_016390) TSPAN15	1/59	1/54 EDI_187220 (196)	1/94 EIN_328370 (199)	1/93 EMO_005920 (143)	1/57 ENU1_177210 (196)	(3?)
EHI_040610 (CL6EHI_040610) TSPAN16	0/–	0/– EDI_013520 (216)	1/96 EIN_152150 (221)	0/– EMO_033580 (222)	0/– ENU1_060540 (213)	–
CL6EHI_198000 TSPAN17	1/59 ^5^	1/60 EDI_157940 (254)	1/41 EIN_371570 (245)	1/50 EMO_050060 (249)	1/59 ENU1_018010 (214)	(2?)

^1^ Based on EHI_139380, ^2^ Based on EHI_168280 (located in the 5’ UTR), ^3^ Based on EHI_152810, ^4^ Based on EHI_107790, ^5^ Based on EHI_198000.

## Supplementary Materials

The following are available online at https://www.mdpi.com/2073-4425/10/11/885/s1: Table S1. List of primer sets used in this study; Figure S1: Multiple sequence alignment of subgroup 1 tetraspanins in *Entamoeba*; Figure S2: Multiple sequence alignment of subgroup 2 tetraspanins in *Entamoeba*; Figure S3: Multiple sequence alignment of subgroup 3 tetraspanins in *Entamoeba*; Figure S4: Predicted secondary structure of introns from representative tetraspanins of subgroup 3; Figure S5: Immunofluorescence assay micrographs of other representative tetraspanin candidates.
