# Supplementary material for: Genome-Wide Analysis of Known and Potential Tetraspanins in Entamoeba histolytica
Source: Genes (Basel). 2019 Nov 3;10(11):885. doi: 10.3390/genes10110885 (PMC6895871; doi:10.3390/genes10110885)
Supplement: Supplementary file 1 [file genes-10-00885-s001.pdf]

Genome-wide analysis of known and potential tetraspanins in *Entamoeba histolytica*

Kentaro Tomii [1,*], Herbert J. Santos [2] and Tomoyoshi Nozaki [2,*]

[1] Artificial Intelligence Research Center, National Institute of Advanced Industrial Science and Technology (AIST), 2-4-7 Aomi, Koto-ku, Tokyo 135-0064, Japan; k-tomii@aist.go.jp

[2] Department of Biomedical Chemistry, Graduate School of Medicine, The University of Tokyo, 7-3-1 Hongo, Bunkyo-ku, Tokyo 113-0033, Japan; hjsantos@m.u-tokyo.ac.jp, nozaki@m.u-tokyo.ac.jp

* Correspondence: nozaki@m.u-tokyo.ac.jp; k-tomii@aist.go.jp

Supplementary Materials

Table S1. List of primer sets used in this study.

**TSPAN_1_1F**	**GTTAGATCTATGAAGGTAATTCAATTCATTGTA**
TSPAN_1_620R	GAAAGATCTAACTGGTGTATATCCTCCAGT
TSPAN_2_1F	GTTAGATCTATGGAAAAACAACGAATATATAACC
TSPAN_2_680R	GAAAGATCTTTGGTCGAGTTTGTTATAACCA
TSPAN_4_1F	GTTAGATCTATGGTTAGTAAACTTGAACTA
TSPAN_4_657R	GAAAGATCTTTCATCTTTGTTATCTCC
TSPAN_9_1F	GTTAGATCTATGGGAGAACATCAACG
TSPAN_9_630R	GAAAGATCTTTCATTATCTGGATCAATAAA
TSPAN_12_1F	GTTAGATCTATGCCATCAACCAAACGT
TSPAN_12_737R	GAAAGATCTTTCCTCAATGTTTTCACTAAACTC
TSPAN_13_1F	GTTAGATCTATGCCATTTGGTATAACA
TSPAN_13_588R	GAAAGATCTTTCCATTAATGGTTCTGT
TSPAN_15_1F	GTTAGATCTATGAACAAAAATCAATCAAAG
TSPAN_15_591R	GAAAGATCTGAATGGTGCTAAATCATC

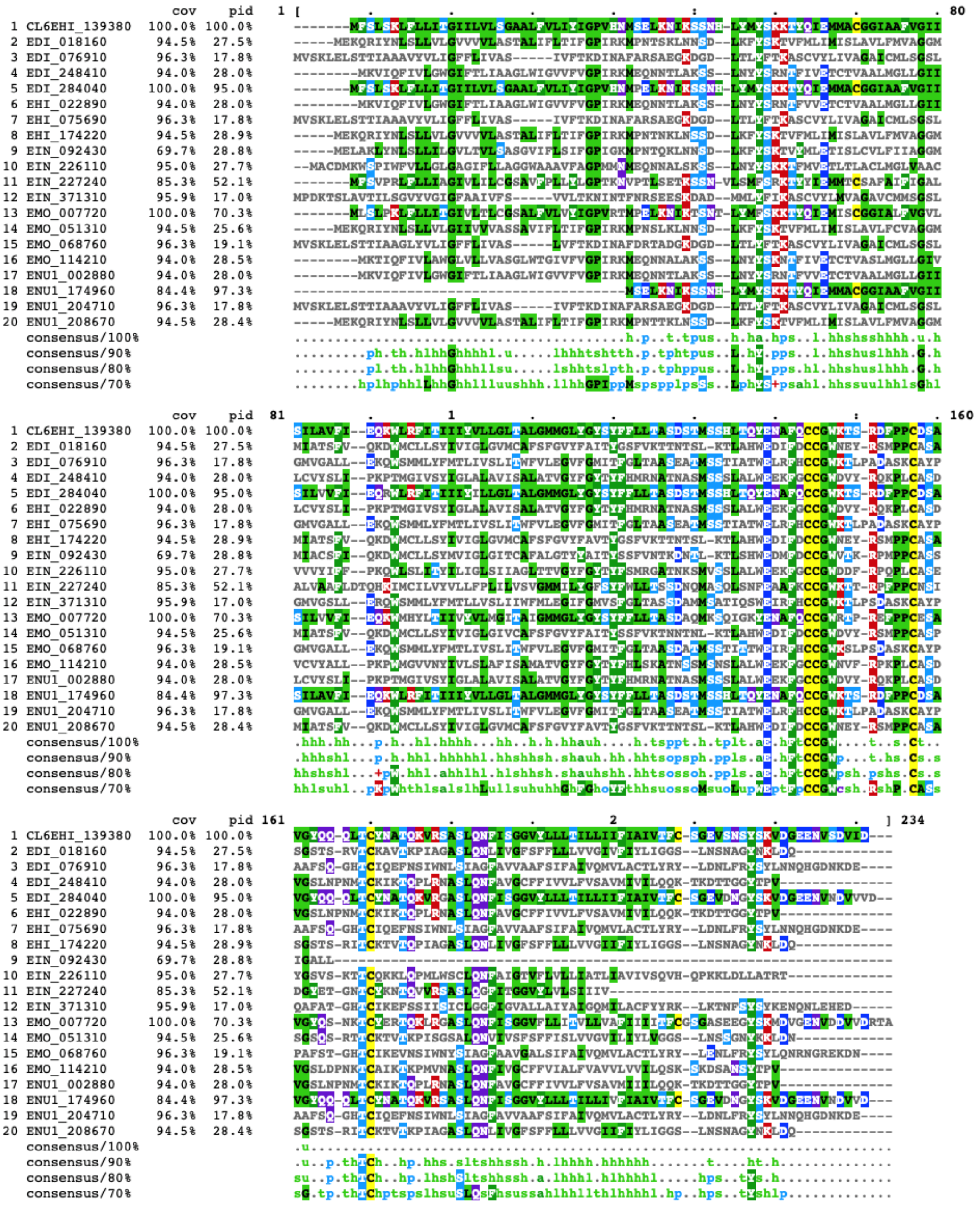

Figure S1. Multiple sequence alignment of subgroup 1 tetraspanins in *Entamoeba*. The figure was created using MView (https://www.ebi.ac.uk/Tools/msa/mview/). Coverage and percentage identity values are indicated in the “cov” and “pid” columns respectively.

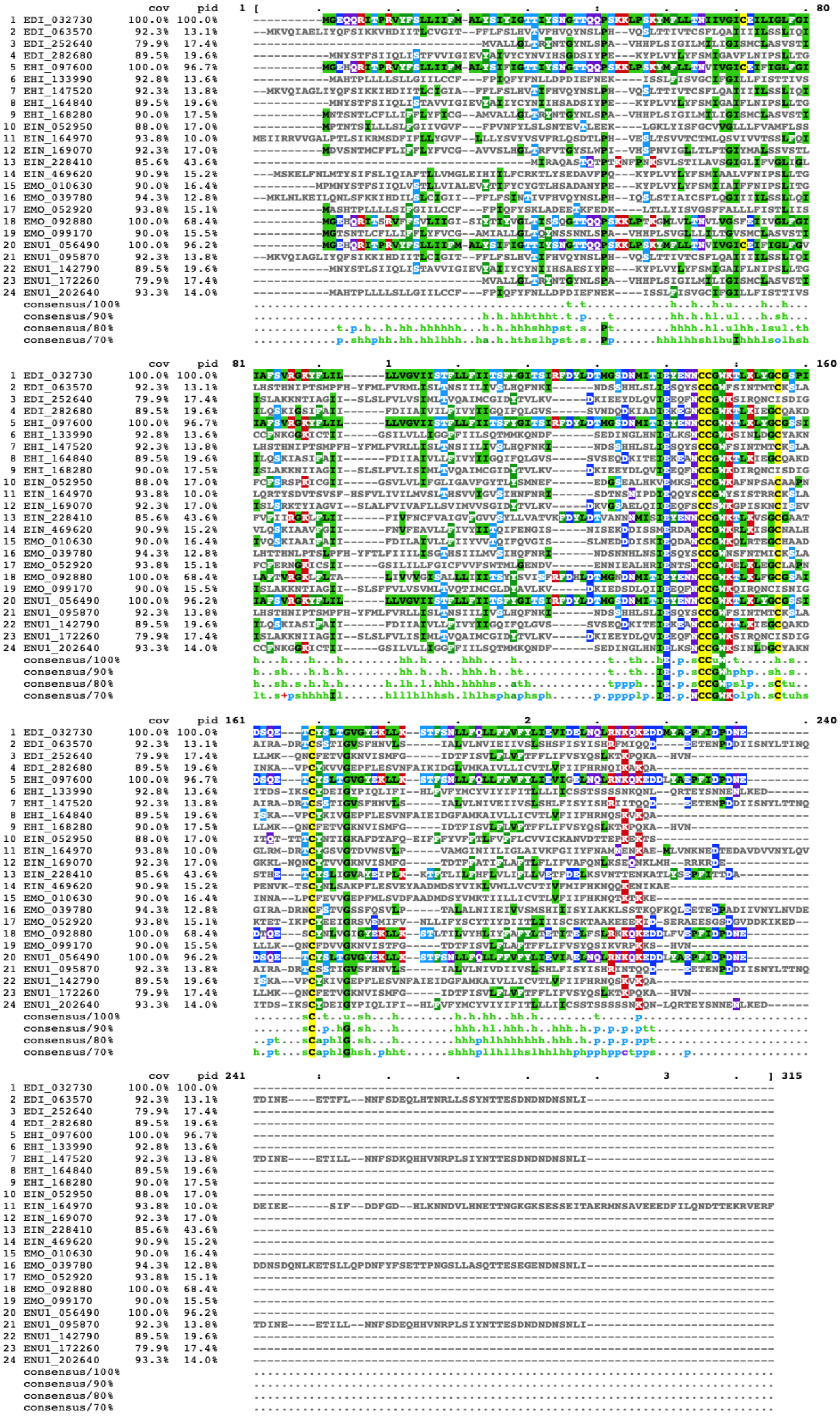

Figure S2. Multiple sequence alignment of subgroup 2 tetraspanins in *Entamoeba*. The figure was created using MView (https://www.ebi.ac.uk/Tools/msa/mview/). Coverage and percentage identity values are indicated in the “cov” and “pid” columns respectively.

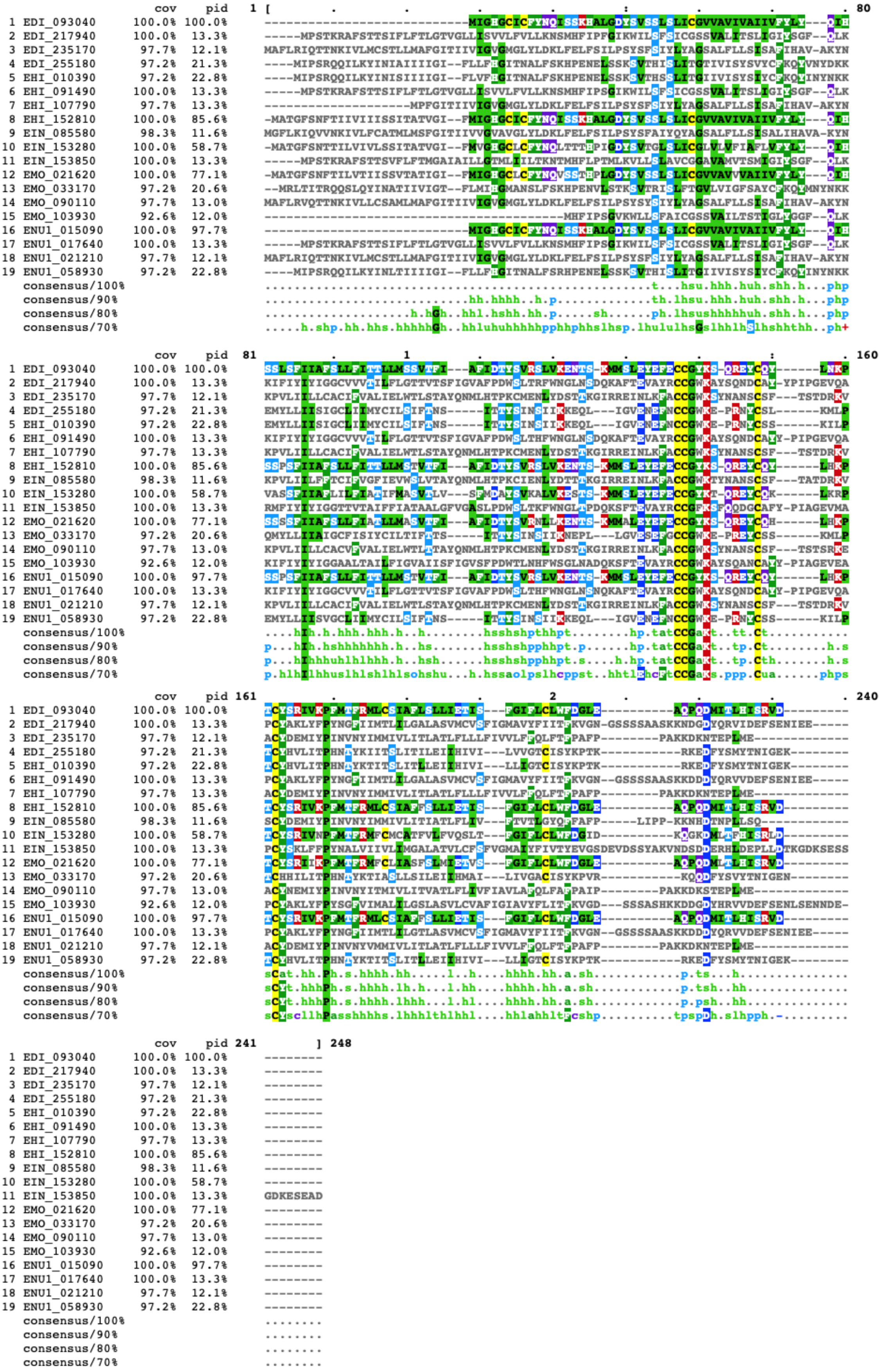

Figure S3. Multiple sequence alignment of subgroup 3 tetraspanins in *Entamoeba*. The figure was created using MView (https://www.ebi.ac.uk/Tools/msa/mview/). Coverage and percentage identity values are indicated in the "cov" and "pid" columns respectively.

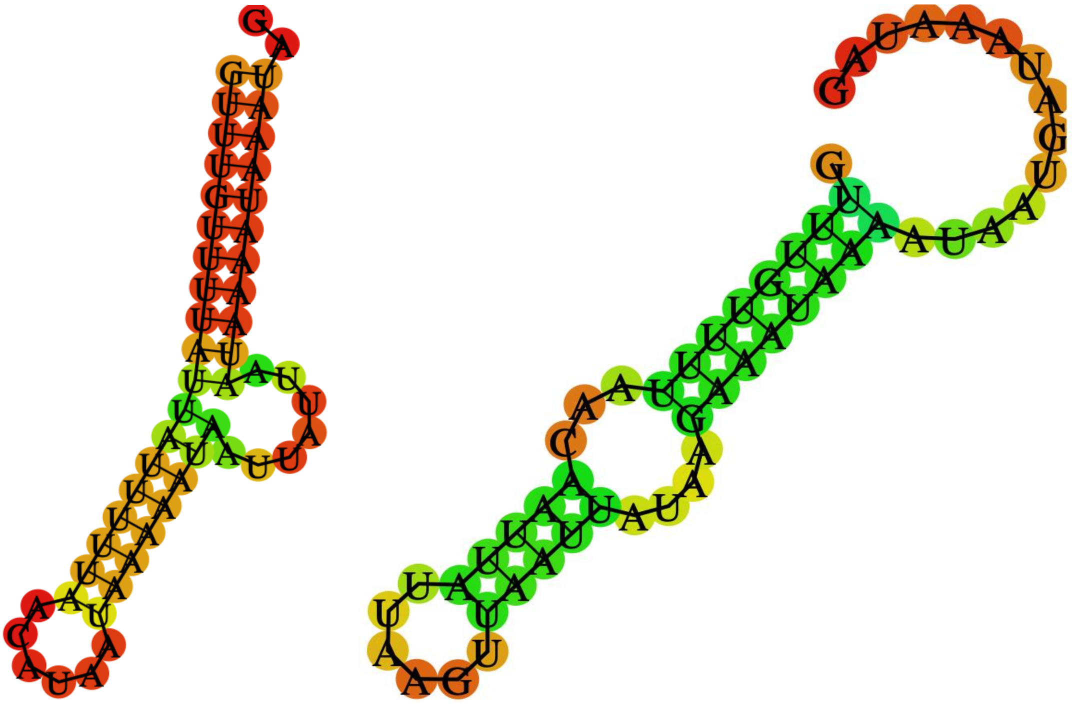

Figure 4. Predicted secondary structure of introns from representative tetraspanins of subgroup 3. Minimum free energy structures for the (TSPAN12) EHI_091490 intron (left) and the (TSPAN13) EHI_107790 intron (right) are depicted. The figures were provided by RNAfold WebServer [29].

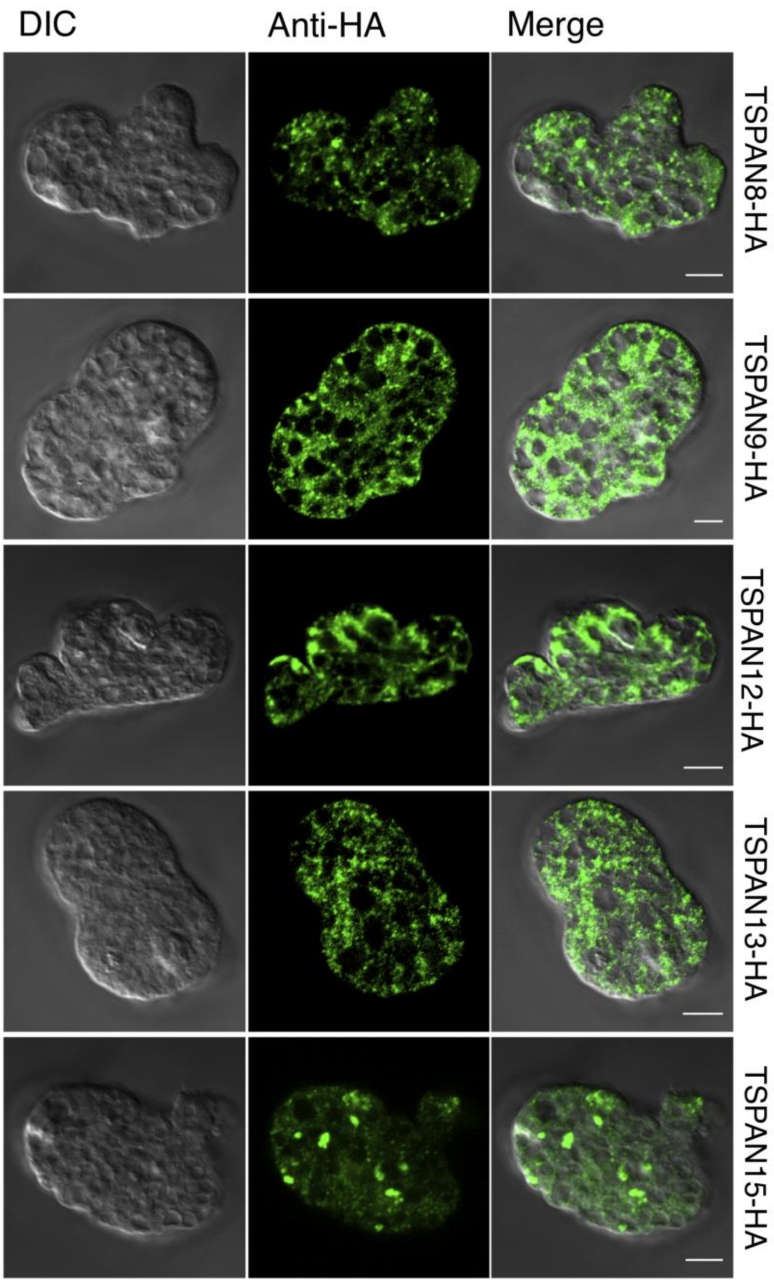

Figure 5. Immunofluorescence assay micrographs of other representative tetraspanin candidates, single-stained with mouse anti-HA antibody (green). Scale bar, 5 μm.
